# “You Got to Keep It Secret”, Barriers to Mental Health Treatment Among Low-income, Midlife Women: A Qualitative Study

**DOI:** 10.1007/s10597-025-01561-x

**Published:** 2025-12-03

**Authors:** S. Tabi, A. Myles, R. Merceir, D. Ore-Onitolo, A. Devlin, S. Fisher, M. F. Morrison

**Affiliations:** 1 Department of Psychiatry, Temple University Health System, Philadelphia, PA USA; 2 Lewis Katz School of Medicine, Temple University, Philadelphia, PA USA; 3 NorthShore University Health System, Research Institute, Evanston, IL USA

**Keywords:** Mental health services, Minority health, Female, Patient preference, Poverty, Health inequities

## Abstract

**Introduction:**

Disparities in mental health treatment for low-income, Black and Latinx populations have been well recognized. Beyond structural barriers, a noteworthy concern was whether attitudinal barriers played a major role in initiating and maintaining treatment. More specifically, 35- to 60-year-old Black and Latina women have been understudied regarding their attitudes and preferences for mental health treatment. The purpose of this study was to identify attitudinal enablers and barriers that have prevented midlife low-income, Black and Latina women from North Philadelphia from initiating and continuing mental health treatment.

**Methods:**

An inductive thematic analysis approach was utilized to inform the sampling, themes, and sub-themes of this study. Semi-structured interviews were conducted with predominantly 50 midlife Black and Latina women from a larger, longitudinal community-based cohort focused on health improvement in North Philadelphia, whose residents were predominantly minority and low-income. Individual semi-structured interviews, with open-ended questions were performed on our study population. This approach stimulated discussion about the participants’ experiences and their feelings that both inhibited and supported accessing mental health treatment. Interviews were conducted, transcribed, and coded to identify themes by the research team. Data analysis was conducted after interviews were coded in 2 research team meetings using flow diagrams.

**Results:**

The participants had a mean age of 50 years old, and the age range was 35–60 years old; most identified themselves as Black (*n* = 37) and reported being unemployed (*n* = 33). Nine overall themes were identified which included considerations of access and sustainability of mental health treatment: attitudinal and structural barriers to treatment, the need for confidentiality, the opportunity to learn coping skills, perceived helpfulness of treatment, medication considerations, therapy as an outlet, prior bad experiences with treatment, and relationships with their mental health professionals. Stigma was influential in discouraging women from seeking mental health treatment. Individual provider-related concerns impacted mental health treatment, including the relationship with the therapist. A prior bad experience with mental health treatment was associated with negative feelings about treatment. Positive feelings about mental health treatment included having an outlet, valuing the relationship with their therapist, and noticing beneficial changes because of treatment.

**Conclusion:**

This study provided deeper insight from the unique community of low-income, primarily Black and Latina women in North Philadelphia. Our findings suggested that efforts to decrease stigma and educate this population of women about the significance and prevalence of mental health disorders may improve the disparities in mental health treatment in this population of midlife women. Continued emphasis on strengthening the connection between the woman and her therapist/psychiatrist and improving access to community-based interventions may help address treatment disparities in midlife women in North Philadelphia.

**Supplementary Information:**

The online version contains supplementary material available at 10.1007/s10597-025-01561-x.

## Introduction

Black and Latina women have faced significant multifaceted barriers in mental health care in the United States (Richards, 2023). Mental health care disparities were partially due to the history of racial discrimination, systemic racism within the healthcare system, socioeconomic disparities, and financial inequity that presented unique challenges for these racial/ethnic groups (Richards, 2023). This long history of negative experiences likely had contributed to feelings of mistrust and stigma associated with the mental health care system.

Most of the literature regarding midlife women focused on White women. Family obligations, career transitions, and hormonal fluctuations associated with perimenopause were some of the common difficulties encountered at midlife (Richards, 2023). Black and Latina women had their own unique experiences with mental health challenges, which resulted in poorer health outcomes compared to white women of the same age (Richards, 2023). Punitive criminal practices in the U.S. produced an environment which discouraged Black people from accessing treatment for fear of detainment. For example, a diagnosis of schizophrenia increased the risk of manual restraints and involuntary admission to an inpatient psychiatric facility (Richards, 2023). With similar clinical presentations, Black people have been three times more likely than White people to be diagnosed with schizophrenia (Richards, 2023; Bodnar-Deren et al., 2017). Fear of detainment could pose as an obstacle for Black women seeking mental health treatment, out of concerns for mistreatment.

There were a multitude of socioeconomic obstacles that contributed to the inequity of mental health treatment in both Black and Latinx populations, including reduced access to mental health treatment, no insurance or inadequate health insurance, “stigmatization of mental illness”, low health literacy, and as mentioned above, the mistrust of healthcare providers (McCall et al., 2023). In cases of women with depression, Black women were about half as likely to receive mental health care compared to White women (Richards, 2023). Younger Black women (median age of 30), with postpartum depression were less likely to accept medications and mental health counseling compared to White women of the same age (Bodnar-Deren et al., 2017). Furthermore, U.S.-born Latinas were more likely to be diagnosed with depression than non-U.S. born Latina women, but less likely to follow up with treatment compared to White women (Temple University Hospital Community Health Needs, 2019). Stigma had been cited as the culprit to these health disparities and persisted in the Black and Latina communities in the North Philadelphia area (Temple University Hospital Community Benefit Report, 2023). Due to stigma, the Latino population with lower income and education were less likely to engage in mental health services and expressed concerns about being “stigmatized by their family and deep concerns about depression medication being addictive” (Lopez et al., 2018). A literature review demonstrated that specifically internalized stigma, the negative belief system that an individual directed to themselves with mental illness, could increase the risk of delayed or avoidance of mental health treatment, poorer social support systems, hopelessness, and increased depression (Vieira et al., 2023). On the other hand, external stigma also affected this population since people with psychiatric conditions were more likely to be discriminated against in relationships and workplaces due to the unfavorable stereotypes and false impressions surrounding mental illness, though it presented differently across cultures (Ahad et al., 2023). A study of Latino patients in a community clinic showed that the more education they received about depression, the less stigma they had about receiving mental health treatment (Lopez et al., 2018).

Our study specifically focused on urban low-income women, primarily Black and Latina, in the midlife age range because this group was understudied and were more particularly vulnerable to depressive symptoms due to a multitude of psychosocial and biological factors. A study demonstrated that older Black women tended to have more severe subclinical depression and were more likely to be “underdiagnosed with depression” in comparison to White women (Erving et al., 2024). This was partially secondary to the social impact of the “Strong Black Woman” concept, which could increase depressive symptoms via emotional suppression and placing others’ needs above their own to overcome systemic injustices and social adversities that they faced on a daily basis (Erving et al., 2024). It had also been postulated that Black Americans’ recurring exposure to socioeconomic hardships could “accelerate the aging process” compared to White Americans, which also could negatively impact the mental health of midlife Black women (Chyu & Upchurch, 2018). On the other hand, the Strong Black Woman concept may also be a protective way to cope with the systemic injustices and racism that they encountered throughout the course of their lives (Erving et al., 2024). Unfortunately, there’s a dearth of research on how the midlife age range uniquely impacted Latina women’s mental health. In our study, we have focused on the unique challenges of Black and Latina women in the North Philadelphia region. Black poverty has been historically an issue in Philadelphia secondary to multiple factors, including housing discrimination involving “racial steering” and “redlining” (Jargowsky et al., (n.d.)). By 1980, poverty levels became more severe and expanded in North Philadelphia from multiple factors including, rapid influx of Latino immigrants, high unemployment rates partially secondary to manufacturing job losses, and “white flight” to the suburbs, thereby deepening the city’s racial and socioeconomic segregation (Jargowsky et al., (n.d.)). North Philadelphia’s population experienced a multitude of social challenges, including lower socioeconomic status, gun violence, high prevalence of substance use, and severe poverty (Temple University Hospital Community Health Needs, 2019). The population was young, the median age was 32 years-old, and over half of the residents were women (Temple University Hospital Community Benefit Report, 2023; U.S. Census Bureau, 2023). North Philadelphia had an ethnic make-up of 35.4% Black and 31.5% Latino (Temple University Hospital Community Benefit Report, 2023; U.S. Census Bureau, 2023). Only 62% of residents living in the area had a high school education, which was substantially lower than the City of Philadelphia (86%) and the United States (89%) (Temple University Hospital Community Benefit Report, 2023; U.S. Census Bureau, 2023). In 2023, approximately 40% of adults in North Philadelphia were under the federal poverty line, compared to a city-wide metric of 23% (Temple University Hospital Community Benefit Report, 2023; U.S. Census Bureau, 2023). Black and Latino people were over twice as likely to live in poverty compared to their white counterparts (Temple University Hospital Community Benefit Report, 2023 (U.S. Census Bureau, 2021). Due to several federal aid programs from the COVID-19 pandemic, the poverty rate in the city of Philadelphia had recently improved, but the challenges remained for those living in poverty to obtain jobs, strive for an education, and find affordable housing (Pew, 2024).

The purpose of this study was to understand the insights and experiences of the predominantly Black and Latina women (ages 35–60 years old) in North Philadelphia when they considered seeking mental healthcare. We explored their perceptions of mental health treatment and their treatment preferences. Ultimately, we acknowledged that the Black and Latina experience in the U.S. was not monolithic, and we have focused on a specific subset of this population that have been uniquely impacted by social and economic adversities in the North Philadelphia region.

## Materials and Methods

The study was reviewed by the Temple University IRB and written informed consent was obtained. Individuals were eligible if they identified as a woman, were aged 35–60 years old, were fluent in English and were enrolled in Temple Health: Block-by-Block (THB3), a community-based research study based out of the academic health care network Temple University Health System. THB3 worked to engage with residents living in Temple University Hospital’s North Philadelphia catchment area. THB3 study participants were recruited via door-to-door canvassing and community events and were asked to complete surveys approximately every six months on a variety of health and behavioral topics to capture longitudinal data focused on health improvement. Complete study methods for the THB3 study sample had been published elsewhere (Fisher & Devlin, 2019).

When individuals were enrolled in THB3, they were also asked whether they would agree to be contacted for other research studies. Participants that met the eligibility criteria were contacted by research staff, whom they had previously met, via phone or in person to ask whether they would be interested in completing a one-time interview to learn more about women’s mental health. Refusals were low but not systematically tracked. Study participants were recruited between February 2017 and April 2018.

### Interviews

Demographic data (collected at THB3 enrollment and updated on a semi-annual basis) were supplemented by a semi-structured interview. Research staff were trained by the Psychiatrist Primary Investigator (MFM) on mental health and the interview guide prior to study initiation. Two female research staff with Master’s Degree level of training and diverse (Black/Latina/White) races/ethnicities attended all interviews. One served as the primary interviewer for most interviews (Black female researcher, with a Master’s degree in social work and substantial clinical experience) and the other served as a recorder. The other research staff did not have mental health training beyond what was provided at the beginning of the study. Most interviews were conducted in a private area of the women’s homes, though some were conducted in research offices to accommodate participant requests. If participants had significant mental health symptoms, they were provided with referrals, if they were interested. Mental health resources were provided to any participants who requested them. Three participants declined to provide consent to be audio recorded. For the non-recorded interviews, detailed notes were taken to capture interview content. Notes were not taken for recorded interviews.

All recordings were transcribed upon completion of the interview. Transcriptions were reviewed by another member of the study team to ensure accuracy. Interview length averaged 75 min and ranged from 25 to 215 min.

### Interview Guide

Two versions of the interview guide were used. One version targeted women who reported mental health struggles at the beginning of the interview, and the other version was used for women who did not have mental health concerns. The guide for women with self-reported mental health struggles included questions regarding previous mental health treatment and concerns while seeking care. Because of the possibility of women to underreport mental health struggles, especially at the beginning of an interview, interviewers were instructed to pivot from one version of the guide to the other if a woman subsequently disclosed mental health concerns and/or treatment. The interview guide developed for this study was provided as supplementary material. Regardless of whether women disclosed a history of mental health struggles, women were prompted to discuss issues such as stigma, positives and negatives of therapy, social acceptance of therapy, and availability of resources in the community. For women who disclosed previous mental health struggles, the interview guide prompted women to discuss their prior experiences with mental health services. Their quotations were identified by an asterisk in the Results (*) section. The study participants were asked to talk about the types of care they had previously sought, along with their current treatment status. If they had discontinued care, they were prompted to discuss the reasons for this decision.

In addition to these open-ended questions, the interview guide also included a variety of quantitative scales, both validated and investigator derived. Transcripts and quotes were from interviews only, not the quantitative scales. These scales covered topics such as caregiving stress, anxiety, depression, loneliness, and trauma. As this portion of the interviews were not included in this study, these scales were not discussed in detail. Participants received a 40-dollar gift card for their participation.

### Qualitative Analysis

**Fig. 1:**
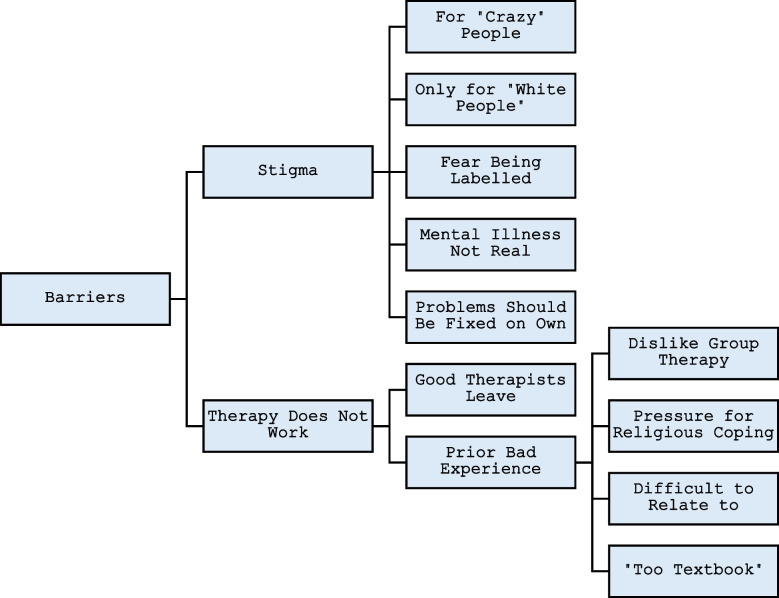
Themes in participants’ perceived barriers to accessing mental health treatment

Inductive thematic analysis informed the themes, and sub-themes of this study. (Braun & Clarke, 2006). No pre-existing theoretical concepts were used for the analysis and transcripts were stripped of identifiers prior to coding. Interviews were pair-coded (meaning coded by 2 of the 5-research staff together in person) utilizing a line-by-line open coding method to identify initial themes by four female research staff (Black (2), White (2)) and one Latino Master’s Degree level male staff member. Coding was performed in one-to-two-hour sessions after clean transcripts were produced (Strauss & Corbin, 1998). The pairs coded five transcripts using thematic analysis that discussed themes and coding, then the pairs were rearranged to prevent coding drifting and unconscious bias. The coding was performed with InVivo 11 software. To ensure coding consistency, group coding meetings were held routinely bi-weekly that included all 5-research staff and a Primary Investigator. The coding and potential themes were discussed and identified in InVivo 11 software. The purpose of these meetings was to identify emerging themes, ensure consistent use of the codes, as well as to resolve any disagreements within the coding pairs. Ten percent of coded interviews were reviewed at the group coding meeting that included all research staff and one of the Principal Investigators (MFM). Pair-coding continued until saturation was achieved for thematic coding. After the open coding was completed, axial coding to identify relationships between themes was performed in 2 research group meetings that were led by a qualitative research expert and included a PI (MFM). Overarching themes for the study and sub-themes were identified from the data collected from the transcripts of individual women. Themes were not checked with study participants after coding. The relationships between the themes and sub-themes were explored using flow diagrams. Figure 1 in the manuscript was the final flow diagram for the study.

## Results

Participants had a mean age of 50.2 ± 8.6 years. Our 50 subjects were predominantly Black and Latina, unemployed women with educational levels of high school and above, who endorsed having mental health struggles. Participants’ ethnicities were reported as primarily 74% Black, 20% Latina, and 6% were non-Latina White or other. 25% identified as single, 18% as having a partner, 20% as married, and 12% as divorced, widowed, or other, as noted in (Table 1). One third of the study participants were employed outside the home. Six out of 50 (12%) of the women did not complete high school/equivalent; 70% reported having previous mental health struggles. In the interview, most of both Black (25 out of 36, 69%) and Latina (9 out of 11, 82%) women reported previous mental health concerns, in response to the first question. Of the three white women, only one endorsed mental health struggles (33%).

**Table 1:** Demographics

Age (years)		
Mean	50.2 ± 8.6	
Range	35–60	
Race		
Non-Hispanic African American	74%	37
Hispanic	20%	10
Non-Hispanic White	4%	2
Other	2%	1
Partner status		
Single	50%	25
Partnered	18%	9
Married	20%	10
Divorced, widowed, other	12%	6
Employment status		
Employed outside the home	34%	17
Not employed outside the home	66%	33
Level of education		
Did not complete high school/equivalent	12%	6
High school graduate	88%	44

The researchers identified nine overall themes resulting from interview coding regarding considerations of access and sustainability of mental health treatment: *structural barriers* – financial, child care support, transportation, and other concerns that impede access to mental health treatment; *confidentiality concerns*- a participant’s fears of discovery within their community that they’re seeking mental health treatment; *learning coping skills*- utilizing coping skills from mental health treatment that they can apply to their daily life stressors; *perceived helpfulness*- a participant’s acknowledgement or perception of the value of mental health treatment in their life, *medication considerations-* overcoming medical mistrust to consider psychotropic medication treatment in addition to therapy; *therapy as an outlet-* therapy as a potential resource for emotional support for psychosocial stressors; *prior bad experiences with treatment*- prior negative experiences with mental health treatment was a perceived barrier to seeking care; *relationships with mental health professionals –* individual meaningful interactions with mental health providers was an influence with staying in treatment; and *provider-related feelings –* participants’ own perceptions or biases towards their mental health providers also was a factor in their engaging in mental health treatment. These 9 themes that reflected the study population’s barriers of seeking mental health treatment are represented in Fig. 1.

Among the concepts, the coding process revealed two particularly prominent themes, the external stigma of seeking mental health treatment, and their individual attitudes towards mental health treatment. Stigma associated with mental health problems and treatment was the dominant social norm interfering with accessing mental health treatment. The belief that mental health care was unhelpful was another critical norm that interfered with accessing care. Individual attitudinal barriers included the idea that people seeking mental health treatment looked unusual or strange, and that seeking mental health care suggested that a person was “weak” or “pathetic”. The relationship with and characteristics of the therapist, and prior bad experience influenced motivation. Some of the other prominent barriers to treatment identified in the interviews are discussed below.

### Barriers to MH Treatment

#### Stigma

Stigma- the negative beliefs held by a society- and the fear of being seen as “crazy” were the most common attitudinal barriers expressed by our study population. This is termed external or public stigma, the negative attitudes expressed toward women with mental health concerns by others, and it influences internalized stigma or one’s personal attitudes towards mental health treatment (Corrigan & Watson 2002). One worry expressed by our study participants was being seen walking into a building demarcated as a site for mental health treatment. These women felt concerned that a passerby would think that something was wrong with them. One Black participant* remarked “I don’t want to go to a place that say, ‘mental health building.” Another Black participant stated “Cause people like me, don’t want no stigma, if I saw one of them in a mental place… It’s your reputation though.” Along the same lines, a third Black woman* remarked:

“Shame, not wanting to go. Thinking somebody’s gonna think that they, something’s wrong. ‘Oh, you’re going to mental health therapy, what’s wrong with you?’ Nothing I just want to make sure what’s right with me. So, I think embarrassment, shame. You know rumors like ‘you crazy’ or something wrong with you.”

If they were seen receiving mental health treatment, these women felt embarrassment and shame if others found out about their treatment. A Latina participant* described her fear of being labeled as someone who had mental health issues:

“…we’ve been so afraid of labels and being ostracized like that… you gotta keep …secret. You can’t let this be known because people not gonna like you. People gonna shun you. Your friends are gonna leave you.”

If a woman was labeled as “crazy” or something similar, she felt there was a strong possibility that she would be cast out of her social community. Given how important a social support network and community was to mental health, women felt that being shunned would be a high price to pay for treatment. The belief that therapy was only for “crazy people” was also a prominent idea. A woman noted that people tended to have difficulty perceiving mental health difficulties as a normal health issue.

Another Black participant* stated “a lot of people won’t do it ‘cause they’re scared that other people will find out. Saying ‘Oh, no, I’m not crazy’. You don’t have to be crazy to see a therapist and people don’t understand that.”

Intense fear of being perceived as “crazy”, then having their reputation harmed and being spurned by their friends and neighbors was a risk women weighed as they contemplated mental health treatment.

#### Perception that People in Therapy do not Look Like Them

A Black participant* thought there should be more emphasis on educating women that people with mental health issues could look just like “normal” people, who were trying to keep a job and cared for their children.

“Yeah, cause coming up when people say ‘my mental health’ it was people like that, like drug addicts, people like the crackheads. It wasn’t like a White actor or like a Black person holding down a family. No, it’s like, people, like, on the streets. And that’s not cool because everybody suffer [sic] from some kind of addiction, you know what I mean? Just like the mental health. It’s not just that Black man who might have that heavy coat on. It could be a White woman with her kids who might have mental health issues. So, if they put that out there. The different kind of, you know, the different people, faces to the mental health list people will be like, ‘Oh, wow.’ Like, I can relate to her. I cannot relate to the guy with the hot coat on, but I can relate to the Black woman who have a job and with kids, who’s been through some s***’.”

Recognizing that mental illness went beyond the stereotypical signs seen in the Black community was a challenge that many Black women faced when considering mental health treatment. This participant’s response suggested that discussing and seeking mental health treatment might be less stigmatized if more women from similar backgrounds and lifestyles openly used therapy without facing negative perception.

A Black woman* remarked on her family member’s belief that therapy was only for “White people”:

“Like I feel like my brother-in-law and his wife they were going through some stuff, and she wanted to go to therapy, and he said blatantly like that’s White people shit I don’t do that we’ll fix it. But…my people they just. They think something is wrong with them like it’s a shameful thing again like it’s embarrassing to them or something. I don’t know…. admitting something’s wrong.”

Furthermore, this woman had a belief that White people could not relate or fully understood the mental struggles of Black women. The utilization of therapy was perceived as a facet of American White culture. In American Black culture, our study participants indicated that people were more reluctant to admit that they needed help from outsiders.

Another Black woman* commented:

“The stigma of saying that you have mental health issues as a Black woman. Um, it’s like you have to champion through a lot of shit. Okay, and it’s like, you’re not allowed to say, ‘Well, the baby’s crying. …. I want to be left alone.’ Like, you can’t say that. And it’s like when people… It’s like, you tell people that you getting help for a mental health disorder it’s like they look at you differently. I know it shouldn’t matter.”

The participants articulated their disinclination to reveal honest emotions in fear of being isolated within their social circles. They felt that engaging in conversations relating to negative emotions could result in them being unfairly categorized as "other" or "different," which could exacerbate their mental health challenges.

#### Internalized Stigma

When stigma was internalized, described as self-stigma or internalized stigma, additional damage could occur. Self-stigma decreased self-esteem, interest in accessing mental health services and diminished hope for treatment benefits. (Volkow et al., 2021).

A Latina participant* noted that people who saw therapists were thought to be pathetic and fragile in her culture. “It’s like a stigma that Puerto Ricans have…You know like ‘She see a therapist? She’s weak.’ Like she cries for anything. Like, stupid”. Not only did she feel pathetic, but this woman perceived that her judgment and intelligence were questioned when she sought outside help. A Black participant* also observed that needing help from a professional signified fragility and said: “… as a weakness. I can’t handle my own, um… I got to go to somebody to figure out what’s going on with me.”

The “Strong Black Woman” was a role that emphasized perceived strength through independence and emotional regulation and inhibited Black women from asking for help (Nelson et al., 2020). This population feared that talking to a professional, a stranger, was a sign of weakness and brought scorn from others.

### Belief that Therapy is Unhelpful

Some women in our study cohort had the idea that receiving mental health treatment demonstrated bad judgment, was unnecessary, and did not help them with their problems. Some study participants felt they did not need therapy, did not believe in mental health diagnoses, and they felt that they should be able to “work it out” on their own. In their social circles, consulting a professional seemed unessential as mental health was perceived to be fiction and a trivial problem. They felt that mental health treatment was a waste of time.

When asked why she did not return to therapy, a Black woman* responded:

“Well, I can just see what they [crying and anger] are myself. You know try to deal with it on my own”.

Some participants described a belief that mental illness did not exist or was inconsequential for their community. Another Black participant* remarked “I don’t even believe in that Bipolar stuff. That’s some new stuff they came up with”. She described Bipolar Disorder as a fad, not a medical disorder with evidence-based treatments. Similarly, another Black woman* shared that no one in her community considered Major Depressive Disorder to be a serious problem. She stated, “Nobody think depression is [real]. It’s not serious.”

While talking about her previous beliefs about therapy being ineffective, a Latina woman* remarked:

“Is people really serious? They go and sit down like dumb asses to take therapy and waste their money?”

She felt that people seeing therapists were fragile, stupid, and squandering their money. There was a perception that mental health disorders were made up so therapists could make money.

### Individual Attitudes: Prior Negative Experiences and Unpleasant Feelings in Therapy

Prior negative experiences with mental health treatment were frequently cited as a factor that prevented women from seeking or continuing mental health care. Some study participants with previous experience in individual therapy wondered whether therapy was beneficial, especially with regards to discussing past trauma. One Black participant* noted:

“I go in one way and come out really like, I don’t know if this helped or not, it’s, you know, like I told you before it’s a very, very touchy and sensitive area for me and I am very emotional when it comes to talking about my past and it’s, it’s hard.” Another Black woman* remarked that therapy was associated with unpleasant emotions, and she didn’t always feel better afterwards. “…we were walking out of the appointments angry, more angry, and in more discord.” A third woman* who was Latina, commented on being unable to recognize any clear benefit, though this may have been due to a short treatment duration:

“No, cause I feel like it don’t work. (laughing) And because it didn’t work first couple times, I’m like, ‘Alright, I’m not trying no more.’

Discussing her past was sensitive for the first Black woman* and therapy was a painful experience for her. The second Black woman* associated therapy with increased anger and discord rather than therapeutic. Finally, a Latina woman's brief experience with therapy led her to believe it was ineffective after a few attempts, which led her to discontinue it. These narratives shed light on the multifaceted, nuanced perspectives that these Black and Latina women had towards the process of therapy.

#### Approach to Treatment: Religious Coping

Some of the women in our study felt pressured by their therapist to use religious coping for stressors. Three women commented on their dislike of being encouraged to use religious coping. A Latina woman* felt as though her preferences were being discounted, saying: “If I needed that I would have gone to a church.” She continued,

“I was seeing another therapist … but I left him because he (Laughter) he said that I was emotionally distressed but it was because I needed spiritual guidance. I was like, what does that have to do with, with whatever I’m going through.”

Religion, offered to the wrong person, could be a strong deterrent to mental health treatment. Offering religious solutions felt like minimization of symptoms to some of the women.

#### Approach to Treatment: Group Therapy

Some study participants voiced a dislike of being pushed into group therapy and expressed that groups of women were not homogenous. They explained that individual women had distinct needs. A Black woman* explained that a group approach hindered the ability of each woman to receive personalized care:

“It’s just, you want me in front of a whole group of people who have so many different other issues, you know, and you going from… It’s, it’s like, alright you want me to fry fish and, uh, but use a popcorn maker.… You understand what I’m saying? It just makes no sense and that made no sense for me to be trying. I didn’t see that you were helping me with all these other things happening in that group.”

This participant* continued, commenting that group members had serious, individual issues and could not all be helped simultaneously. She also expressed concerns that some women may feel overwhelmed by the problems and emotions of other group members:

“If you get, to me, if you get a group of people that are on the same page, that’s one thing. But you get, like, like you’re going with. You’re bipolar. Okay, you’re schizophrenic. I’m psychotic. So, you got one person saying, “Oh, I can’t handle this from her. I can...” No, the hell you can’t. Deal with one issue at a time. How you going to have all three of us, with all three different spectrums and you’re going to say you’re helping. I don’t think so. ….In my mind’s eye, it just wasn’t working, you know. Put me with somebody that, that has walked in my shoes or at least got them on, you know what I’m saying?”

Although group therapy was a popular, evidence-based method of therapy, some women in our study cohort believed that this therapeutic method did not work for everyone. For this study participant, she felt that personalized individual therapy was likely to be more effective.

#### Treatment: Relationship with the Therapist

One of the prominent themes expressed by our study cohort as an important factor in therapy was a strong connection to the therapist. The bond they felt with their therapist was critical in these women’s decisions to stay in treatment. Several complaints about therapists’ styles emerged from our study participants, such as a dislike of therapists or doctors who were “too textbook” (hard to relate to or arrogant) and was a factor in women ending mental health treatment prematurely or preventing reestablishment of services. A Black participant* stated,

“He just was not the right person, and he was more like… He was, he was…, to me more like a damn bully. Cause like, you going, you damn well going to get this out of me and I’m like, “Oh no, you don’t know me very well”. I can put a S on stubborn with a quickness. He just wasn’t right for me.”

A respectful relationship was emphasized by our study population as critical to treatment success. Doctors or therapists perceived as judgmental negatively influenced women’s experiences with treatment. One Black participant* remarked:

“Some people they get airs, put on airs and act like they better than other folks. You can’t help somebody heal if you think you are better than them. … Who gonna trust you?”

(How long did you see that therapist?) “One day. I don’t know, I didn’t even stay. I didn’t stay because you already done, diagnosed me, pre-diagnosed, you pre-diagnosed me before I even came in the room. You know, you didn’t even hear what happened, or what I was going through, or the possibilities of me going through anything in the future. You, you didn’t know anything about me. Except for what was told to you. And for you to, to judge and to diagnose everything? You couldn’t. You couldn’t.”

This woman felt pre-judged, not listened to and not understood. She realized that she didn’t trust the therapist and subsequently terminated the therapy services.

Some study participants questioned the motives of the doctor or therapist and worried that the provider was only in it for the money. A Latina woman* stated, “The doctors today they just want you just sit there, you talk, they prescribe, and let’s go. There’re not many doctors that want to do their real profession. They’re just in for the money, in for the time.” The woman* was also worried that her therapists were unskilled and worried about their training, “I mean a lot of people shouldn’t be giving therapy if they don’t have the proper education”. Another commented, “I don’t want to get a therapist that’s really just like there because it’s their job and I’m going to do my eight and skate [9–5 job].” Unskilled therapists, dubious motives, an unprofessional atmosphere, and feeling judged by the therapist were separate and prominent themes that influenced these women’s decisions regarding treatment.

A Black participant* expressed a sense of loss and lack of direction after her therapist, whom she felt comfortable with, left and discontinued the treatment:

“So that’s what I kind of, like, related it to, but then she helped me open up where I was able to talk to her. And I had gotten comfortable with speaking with her because this was the only person that I spoke with. You know, and I got comfortable opening up to her. But then after she left, it’s like, what do I… Where do I go from here?”

Despite establishing a strong connection with their therapists, a few of our study participants found the prospect of ending their mental health treatment to be an unsettling experience. The sudden loss of communication, with no alternative treatment in place, was sometimes a significant barrier preventing women from continuing mental health care.

#### Positives about Mental Health Treatment

These women found therapy to be helpful in both understanding their past adverse experiences in childhood and coping with their current family, community and health challenges. Having an outlet for their feelings felt supportive to them. A few women in our study felt that therapy “worked” and were pleased with gains and success with treatment. They valued their relationship with a therapist/psychiatrist. Some traits that this population appreciated in a therapeutic relationship with their mental health provider included: 1) liking a therapist who was confident, skilled, or genuine; 2) of a similar background to the patient; 3) could relate to the patient’s struggles; and 4) was a good listener.

## Discussion

This study was unique because it was the first qualitative study to explore the perceptions and barriers to mental health treatment of low-income women of color in the midlife age group. It was one of the few pieces of scholarship which examined barriers to mental health care in a substantial group of midlife Black and Latina women in an underserved area using qualitative methods. Through interviews, our participants shed light on several factors that influenced their mental health care utilization, as well as views on stigma, treatment preferences, and prior experiences with mental health care. Other studies have explored barriers to mental health treatment of low-income women in rural settings, immigrant women, and mental health systems in low-income countries, but no other studies focused on low-income, midlife women of color (Snell-Rood et al., 2017; Lempp et al., 2018).

The Black and Latina women of North Philadelphia have been historically overlooked, and resource-limited community settings could hinder access to mental health treatment and negatively impact women’s satisfaction with mental health providers. This study was unique because it was the first qualitative study to explore the perceptions and barriers to mental health treatment of low-income women of color in the midlife age group. It was one of the few pieces of scholarship which examined barriers to mental health care in a substantial group of midlife Black and Latina women in an underserved area using qualitative methods. Through interviews, our participants shed light on several factors that influenced their mental health care utilization, as well as views on stigma, treatment preferences, and prior experiences with mental health care. Other studies have explored barriers to mental health treatment of low-income women in rural settings, immigrant women, and mental health systems in low-income countries, but no other studies focused on low-income, midlife women of color (Snell-Rood et al., 2017; Lempp et al., 2018).

Among the barriers highlighted in this study, stigma was the most significant contributor to non-utilization of mental health care services. External stigma, or the negative perception held by a society, had been widely cited as a factor that impacted all people from utilizing mental health care, and this was strongly felt by our participants. Many of our study participants expressed fear in seeking treatment due to concern for their reputation and not wanting to seem “crazy” to their peers. Similarly, rural white Appalachian women were concerned about being perceived as “crazy”, so this fear was not limited to North Philadelphia women of color (Snell-Rood et al., 2017). One Latina participant* feared being ostracized and shunned, consistent with findings that higher rates of people in Latinx communities experienced feelings of shame and embarrassment towards mental health problems, further preventing them from seeking treatment (Pérez-Flores & Cabassa, 2021). A Black participant expressed fear of appearing “weak” to others, indicating that part of the stigma around treatment also included a perception of fragility. Connected to that was an expectation in the Black community for women to be independent and not ask for help. Previous research demonstrated an inverse relationship between mental health stigma and treatment-seeking in both Black and Latino communities, which emphasized the need to further explore this shared attitude towards mental health in these populations (Fripp & Carlson, 2017). The intersectionality of Black and Latina women, who often faced financial and other structural barriers, contributed to compounded complex barriers to their access of care.

A prior negative experience in therapy and/or dissatisfaction with a previous mental health provider was a key barrier that prevented our study population from seeking treatment again. Lack of a meaningful connection with their mental health care provider was another hurdle to treatment engagement. These women noted they were unable to mesh with their therapists’ styles, felt “bullied”, and/or judged, which led them to discontinue therapy. This perception of being judged or misunderstood by the therapist may be secondary to internalized stigma, underlying mistrust of healthcare providers, and/or consideration of culturally sensitive mental health treatment. Low-income Appalachian women similarly reported that “poor treatment quality-not merely access” was a major impediment in their seeking mental health treatment (Snell-Rood et al., 2017). Notably, confidentiality was not expressed as a concern by our participants whereas one Appalachian woman stated, “everyone that goes there gets talked about”, and confidentiality was a significant worry for rural women in small communities (Snell-Rood et al., 2017). Also, cost was noted to be the most common reason Black adults did not seek mental health treatment in a recent review but was not mentioned by our study participants as a barrier to treatment (Ruth Shim, 2021).

A study showed that although Black women were more likely to experience depression compared to White women, they were less likely to utilize mental health treatment (Poleshuck et al., 2013). In our study, many Black women remarked that mental health treatment “wasn’t right for them”, which may be secondary to the stigma and sense of alienation from their community if they were engaged in mental health treatment. These findings were consistent with another study that explored barriers to psychotherapy in low-income Black women, and they shared similar sentiments that the process of therapy was invasive and a violation of one's privacy (Poleshuck et al., 2013). A few of the frustrations with treatment expressed by the North Philadelphia women were also expressed by a study of White rural women in Appalachia, suggesting that approaches to improving mental health treatment may be adaptable to other populations of low-income women (Snell-Rood et al., 2017). Even perceived discrimination could cause increased levels of stress and lead to nonparticipation in healthy behaviors or seeking mental health treatment (Pascoe & Smart Richman, 2009).

### Future Recommendations

The results of this study emphasized the importance of increased study of low-income Black and Latina women in underserved communities and identified ways to reduce barriers to available resources for mental health treatment. Further research and interventions should focus on improving patient-provider relationships, and reducing stigma, which may improve treatment utilization. Strong therapeutic alliance between the patient and provider was associated with increased patient satisfaction, adherence to pharmacological treatment and keeping appointments (Stanhope et al., 2013). Kaiser Permanente, an integrated managed care consortium, had adapted a model of addressing patient satisfaction by continuously assessing feedback about treatment, including the therapeutic alliance. Therapeutic alliance was assessed using the following criteria: agreement on goals, treatment plan, and mutual understanding and respect, then the results were discussed with the clinician(Pascoe & Smart Richman, 2009). This type of model may be a way to improve the connection and shared decision-making between the patient and clinician.

Previous research had shown that Black people have reported preferring racially concordant provider-patient relationships (Lin, et al., 2018; O’Malley, 2021). This may help optimize cultural competency in mental health care which could incentivize treatment utilization within the Black community. However, only 2% of psychiatrists were Black and only 4% of psychologists were Black in the United States (Lin, et al., 2018; O’Malley, 2021). Furthermore, 10.8% of psychologists and 9.5% of psychiatrists identified as Latino in the United States (Psychologist Demographics and Statistics, 2023; Psychiatrist Demographics and Statistics (2023)). Initiatives to increase under-represented Black and Latina women in Psychiatry and other mental health professions would continue to be important in providing and optimizing treatment for Black and Latina women.

Our participants shared that they would like to be part of a community board with input into the mental health of the community as a way of improving mental health treatment. Other options included community-based interventions that ‘meet patients where they are at’ through involvement of community leaders and utilization of non-stigmatizing settings, such as churches or schools, as sites to provide mental health care. This type of intervention could help reduce stigma while simultaneously reducing other barriers such as transportation, health insurance, etc. A recent research study demonstrated that there was a significant reduction of “both public and self-stigma” and increased positive attitudes towards seeking mental health treatment after community-based mental health services had been introduced (Kearns et al., 2019). Contact-based intervention is another modality that aimed to provide a forum for Black and Latina women to hear first-hand stories from similar women with mental health diagnoses. These types of interventions could be the most impactful approaches in diminishing stigma in these marginalized populations (Conner et al., 2023).

### Limitations

While this qualitative study had a robust sample size, this was an exploratory study, conducted with a community-based convenience sample from a larger longitudinal study in North Philadelphia. Though individual interviews were conducted, most of the data reflected the feelings of women who endorsed mental health struggles. A different format may be required to get more information from women that may have psychiatric disorders but are reluctant to voice them in an interview setting. In addition, the findings may not be generalizable to other populations that are low-income, urban, midlife women of color, such as Asian, American Indian, and Bi/Multi-Racial women, who were not represented in our study population. The 3 white participants, 2 of whom did not endorse mental health struggles, did not provide information about mental health barriers.

## Conclusion

Addressing attitudinal barriers such as stigma, minimization of mental health problems, culturally competent mental health treatment, and medical mistrust could increase utilization of mental health treatment services of this population. We recommend future research and interventions to improve therapeutic alliance, increase culturally competent care, and reduce stigma. The mental health of low income midlife Black and Latina women who live in urban settings had been ignored with detrimental results to women and their communities. This study provided deeper insight from the unique community of low-income, Black and Latina midlife women in North Philadelphia.

## Supplementary Information

Below is the link to the electronic supplementary material.

Supplementary file1 The interview guide developed for this study was provided as supplementary material. In addition to questions regarding previous mental health treatment and concerns while seeking care, the interview guide included a variety of quantitative scales, both validated and investigator derived. These scales covered topics such as caregiving stress, anxiety, depression, loneliness, and trauma. (PDF 286 KB)

## Data Availability

Inquiries about access to de-identified data can be made to the senior author, Mary F Morrison, MD, MS, co-P.I.

## Declarations

### Positionality Statement

As a group of academic women clinical researchers composed of psychiatrists (2), medical students(2), an epidemiologist with a nursing background, research assistants, identifying as both white (3–4) and black women (3), we acknowledge the dimensions of our identities that shape our work. Our understanding of mental health and treatment is shaped by our formal education and experiences with patients in our community of North Philadelphia We are aware that psychiatry is a field intertwined with power dynamics, historical inequities, and stigma. We work to challenge our biases, especially in the context of race, ethnicity and socioeconomic status, and recognize disparities in mental health care that can affect marginalized populations. As we engage with patients and conduct research, we strive to provide culturally competent care and contribute to efforts to eliminate systemic barriers in the field. We are committed to having the voices and experiences of midlife women in North Philadelphia heard and better understood through this study and we honor their contributions.
