# Supplementary material for: “You Got to Keep It Secret”, Barriers to Mental Health Treatment Among Low-income, Midlife Women: A Qualitative Study
Source: Community Ment Health J. 2025 Dec 3;62(4):692–704. doi: 10.1007/s10597-025-01561-x (PMC13144273; doi:10.1007/s10597-025-01561-x)
Supplement: Supplementary file 1 — Supplementary file1 The interview guide developed for this study was provided as supplementary material. In addition to questions regarding previous mental health treatment and concerns while seeking care, the interview guide included a variety of quantitative scales, both validated and investigator derived. These scales covered topics such as caregiving stress, anxiety, depression, loneliness, and trauma. (PDF 286 KB) [file 10597_2025_1561_MOESM1_ESM.pdf]

HH Code: ________

Participant ID: _____

Staff Initials: _______

Interview Date: ____

Start Time: _______

End Time: ________

For THB3 staff use only

Women's Mental Health – Interview Guide

By talking with you today, we want to better understand your needs and experiences. Some of the topics may be sensitive and we will jump from topic to topic. Please feel free to expand as much as you feel comfortable. Sometimes we will ask you more questions about an idea or topic, we do this to understand what the topic or experience means to you.

We will be taking notes throughout the interview so please don't let our notetaking disturb your train of thought.

When we say "struggled with mental health" we are referring to any feelings or behaviors that you might have that bother you or get in your way. Some example of these types of behaviors that people often talk about are eating secretly, crying, or feeling your heart race. These are just some examples.

Packet #:____________

Section A: _For Women who have previously struggled with their mental health_

1. **Recently (past 2 weeks), what mental health problem has been bothering you the most, if any?**
   (If participant says there are no mental health problems, move to Section B)
   Probes: * What makes this problem the worst? * How is this problem affecting your life? * How does this problem make life difficult? * How does this problem affect your stress level? * How does this problem interfere with your life? How often (interferes)? When?
   [Clarify MH versus other issues/problems -- If a problem named is not a mental health issue, ask "How does that (MH problem) make you feel?" If after three tries there is still a lack of disclosure of feelings or behaviors related to mental health, give examples of mental health.]

2. **How old were you when you began struggling with that problem?**
   _____<18 years_______18-34____35+ _____Unknown/Refused

If these experiences come up:

- _Physically abused or punished:_
  - _At what age? _______________
  - _How many times? 1 / 2-4 / 5-10 / more than 10_
- _Seriously injured, or feared that you might be seriously injured:_
  - _At what age? _______________
  - _How many times? 1 / 2-4 / 5-10 / more than 10_
- _Attacked by a gun, knife, or some other weapon?_
  - _At what age? _______________
  - _How many times? 1 / 2-4 / 5-10 / more than 10_
- _Seen someone seriously injured or killed:_
  - _At what age? _______________
  - _How many times? 1 / 2-4 / 5-10 / more than 10_
- _Intercourse or oral or anal sex against your will?_
  - _At what age? _______________
  - _How many times? 1 / 2-4 / 5-10 / more than 10_
- _Touched private of your body:_
  - _At what age? _______________
  - _How many times? 1 / 2-4 / 5-10 / more than 10_
- _Emotionally abused or neglected:_
  - _At what age? _______________
  - _How many times? 1 / 2-4 / 5-10 / more than 10_
- _Ever live without at least one of your biological parents?_
  - _At what age? _______________
  - _For how long? _______________

3. Is that the only mental health problem you have or do you think you have others?

(Give time to talk about other MH problems, use guidance above to probe how MH problem affects life)

*(Mark off items that were mentioned previously, then read off the remainder of the list. Mark answer if they have **struggled** with the problem, even if they have not had a formal diagnosis)*

___ Anxiety disorder

Circle if known: Generalized Anxiety Disorder / Panic Attacks / OCD / Hair Pulling / Skin Picking

___ Mood disorder (depression, bipolar disorder)

___ Post-traumatic stress disorder or severe reaction to life threatening experience, like physical or sexual assault

___ Memory Problems

___ Attention Deficit Hyperactivity Disorder (ADHD)

___ Suicidal Feelings or Attempts

___ Schizophrenia or Hearing Voices that aren't there for hours at a time

___ Eating disorder (binge-eating disorder, bulimia, anorexia or another type of disorder (eats unusual things like dirt etc.)

*(If indicates Addition or Substance Use Disorder, **read** options below and check all that apply)*

___ Addiction or Substance Use Disorder

___Alcohol
___Marijuana
___Prescription pills, either own pills, other peoples, street pills[types, names)
___Heroin
___Cocaine
___PCP
___Other (methamphetamine, LSD__________)

___ Bereavement (close family member/close friend dying) *(If the participant has experienced a loss, probe to see how the loss is affecting them and their mental health.)*

How many and who? *(Put most recent first)*

___Other_______________

Section A: *For Women who have previously struggled with their mental health*

4. **Have you received treatment for that problem/those problems?** *(Use shaded box below for comments)*
___Yes *(ask questions 4a, 4b, 4c, and 4d)*
___No *(move to question 5d)*

4a. What treatments have you tried?
(Allow participant to respond – then read remaining options after initial response)
______Medication
______Individual therapy
______Group therapy/Support groups
______Hospitalization
______Substance abuse treatment
______Other (light therapy, ECT etc.)
______ Unknown/Refused

4b. How was treatment helpful? Why or why not? *Probes: * What worked? What was it like to experience that? * What didn't work? * What was challenging or difficult about treatment?*

4c. What can you think of that would have made your treatment work better for you? *Probes: How could treatment be helpful to you? What would that look like?*

4d. Did you stop treatment? ___Yes *(ask Question i)* ___ No *(ask Question ii)*

i. If yes, reasons why? *[treatment stopped]*
Probe: If participant gives multiple reasons, ask for the primary reason.

(After interview, indicate which theme was indicated)

___Stigma, embarrassed, didn't want others to know
___Bad experience before
___Couldn't get an appointment-gave up
___Don't want medication
___Too inconvenient
___Wanted to handle it on my own
___Worried about cost
___Other (specify): ____________________________

ii. If no, why not? *[treatment continued]*
Probe: What was it about the treatment that helped your stick with it / stay?

5. *Check off topics as they are addressed naturally during interview – ask remaining questions:*

____ *Positive/Negative Views on therapy:* **What do you think about therapy (for emotional or mental support)?**

____ *Stigma:* **How do you feel about people who seek therapy?**

____ *Social Acceptance of Therapy:* **What would you recommend to a friend who you believe is experience pressures that are beyond typical?**

____ *Availability of Resources in the Community:* **What mental health resources/services do you feel that may be beneficial to the women in your community?**

***Section A:** For Women who have previously struggled with their mental health*

6. **When was the last time you were feeling good, like yourself, for at least a week?** *Probes: What were the circumstances (most recently)? What was happening? What were you doing? What did it feel like? (If participant says they "never feel good" note that.)*

7. **What do you do for yourself now to make yourself feel better? How often do you do that?** *Probes: What have you done to make yourself feel better? What are you doing that makes you feel better emotionally?*

8. **Do you look forward to a happy future?** *(Use shaded box below for comments)*
   ___Yes ___ No

9. **What <u>would you want</u> your future to look like ten years from now?** *Probes: What would you like to be doing in 10 years? Where would you like to be living? Who would be in your life?*

Section B: *For Women who have <u>not</u> previously struggled with their mental health*

1. **When feeling stressed or overwhelmed, what do you typically do to decrease it?** *Probes: What is your experience? What makes it difficult to decrease feeling stressed or overwhelmed?*

b. **If you would, share with me a time when you were fed up with everything.** What happened? What did you do to get through it?

2. **What types of community activities are you involved in?** *Probe for engagement: What do you do? What is your role? How often? What is it like?*

3. **What do you do for yourself now to make yourself feel better? How often do you do that?**

4. *Check off topics as they are addressed naturally in interview – ask remaining questions:*

____ *Positive/Negative Views on therapy:* **What do you think about therapy (for emotional or mental support)?**

____ *Stigma:* **How do you feel about people who seek therapy?**

____ *Social Acceptance of Therapy:* **What would you recommend to a friend who you believe is experience pressures that are beyond typical?**

____ *Availability of Resources in the Community:* **What mental health resources/services do you feel that may be beneficial to the women in your community?**

Section C: _For <u>all</u> Women_

Part 1 – Please read each question and corresponding responses slowly

1. **A caregiver is someone who is responsible for others. This could include caring for family or non-relatives. This could be helping care for someone's life or financial, logistical, emotional or physical wellbeing. Are you presently a caregiver?** _(Use shaded box below for comments)_
   ___Yes _(move to questions 1a and 1b, and complete this section)_
   ___No _(move to the Part 2)_

   1a. If yes, for whom?____

   1b. Age(s)________________________

2. **Are there downsides of your caregiving role?**
   ___Yes _(move to question 2a)_

   ___No

   2a. What? _(Probe for negatives of caregiving role: what is it like to be in this role?)_

3. **Are you satisfied with the support your family has given you?**
   ___Yes
   ___No

4. **On a scale of 1 to 10, with 1 being "not stressful" and 10 being "extremely stressful," how would you rate your current level of stress?**____ _(Probe for levels of stress: how does that level feel like?)_

***Section C:** For <u>all</u> Women*

5. **Do you feel stressed between caring for your relative and trying to meet other responsibilities (work/family)?** (*Read options)*

___0=Never

___1=Rarely

___2=Sometimes

___3=Quite Frequently

___4=Nearly Always Has

6. **Do you feel an overall feeling of burden from caring for your relative?** (*Read options)*

___0=Never

___1=Rarely

___2=Sometimes

___3=Quite Frequently

___4=Nearly Always Has

Section C: *For all Women*

Part 2 - Read: The questions in this scale ask you about your feelings and thoughts during the last month. In each case, please indicate how often you felt or thought a certain way.

1. In the last month, how often have you felt that you were unable to control the important things in your life? (*Read options)*

___0=Never

___1=Almost Never

___2=Sometimes

___3=Fairly often

___4=Very often

2. In the last month, how often have you felt confident about your ability to handle your personal problems? (*Read options)*

___0=Never

___1=Almost Never

___2=Sometimes

___3=Fairly often

___4=Very often

3. In the last month, how often have you felt that things were going your way? (*Read options)*

___0=Never

___1=Almost Never

___2=Sometimes

___3=Fairly often

___4=Very often

4. In the last month, how often have you felt difficulties were piling up so high that you could not overcome them? (*Read options)*

___0=Never

___1=Almost Never

___2=Sometimes

___3=Fairly often

___4=Very often

Section C: *For all Women*

Part 3 – Read:

1. **In the past month, have you had any problems concentrating on what you were doing?**
   ___Yes *(move to question 1a)*
   ___ No *(move to question 2)*

   1a. If yes, how many days in the past week did you notice problems with your concentration?
   __0
   __1-2
   __3-4
   __5-6
   __7

2. **In the past month, have you had any problems with forgetting things?**
   ___Yes *(move to question 2a)*
   ___ No *(move to question 3)*

   2a. If yes, how many days in the past week did you notice problems with your forgetting things or your memory?

   __0
   __1-2
   __3-4
   __5-6
   __7

Part 4 – Read:

1. **Do you currently smoke tobacco on a daily basis, less than daily, or not at all?**
   ___ Daily=1
   ___ Less Than Daily=2
   ___ Not At All=3
   ___ Unknown=4

2. **(Ask only if participant says they do not currently smoke tobacco): Have you smoked tobacco daily in the past?**
   ___ Yes
   ___ No

***Section C:** For <u>all</u> Women*

Part 5 – Read: The following statements describe how people sometimes feel. *(Read options after each question)*

1. **How often do you feel that you lack companionship?**
   ___1=Never
   ___2=Rarely
   ___3=Sometimes
   ___4=Always

2. **How often do you feel that there is no one you can turn to?**
   ___1=Never
   ___2=Rarely
   ___3=Sometimes
   ___4=Always

3. **How often do you feel alone?**
   ___1=Never
   ___2=Rarely
   ___3=Sometimes
   ___4=Always

4. **How often do you feel that there are people you can talk to?**
   ___1=Never
   ___2=Rarely
   ___3=Sometimes
   ___4=Always

5. **How often do you feel close to people?**
   ___1=Never
   ___2=Rarely
   ___3=Sometimes
   ___4=Always

6. **How often do you feel that there are people you can turn to?**
   ___1=Never
   ___2=Rarely
   ___3=Sometimes
   ___4=Always

7. **How often do you feel left out?**
   ___1=Never
   ___2=Rarely
   ___3=Sometimes
   ___4=Always

8. **How often do you feel isolated from others?**
   ___1=Never
   ___2=Rarely
   ___3=Sometimes
   ___4=Always

***Section C:** For <u>all</u> Women*

Part 6 – Read:

Over the last 2 weeks, how often have you been bothered by the following problems?

1. **Feeling nervous, anxious, or on edge?** (*Read options)*
___0=Not at all
___1=Several days
___2=More than half the days
___3=Nearly every day

2. **Not being able to stop or control worrying?** (*Read options)*
___0=Not at all
___1=Several days
___2=More than half the days
___3=Nearly every day

(If above score ≥ 1 continue with next 5 questions)

3. **Worrying too much about different things?** (*Read options)*
___0=Not at all
___1=Several days
___2=More than half the days
___3=Nearly every day

4. **Having trouble relaxing?** (*Read options)*
___0=Not at all
___1=Several days
___2=More than half the days
___3=Nearly every day

5. **Being so restless that it is hard to sit still?** (*Read options)*
___0=Not at all
___1=Several days
___2=More than half the days
___3=Nearly every day

6. **Becoming easily annoyed or irritable?** (*Read options)*
___0=Not at all
___1=Several days
___2=More than half the days
___3=Nearly every day

7. **Feeling afraid as if something awful might happen?** (*Read options)*
___0=Not at all
___1=Several days
___2=More than half the days
___3=Nearly every day

Section C: *For all Women*

Part 7 – read options after each question:

Not at all=0 Several days=1 More than half the days=2 Nearly every day=3

Over the last 2 weeks, how often have you been bothered by the following problems?

1. Little interest or pleasure in doing things? ____
2. Feeling down, depressed, or hopeless? ____

If score ≥ 1 continue with next 5 questions.

3. Trouble falling asleep, staying asleep, or sleeping too much? ____
4. Feeling tired or having little energy? ____
5. Poor appetite or overeating? ____
6. Feeling bad about yourself – or that you're a failure or have let yourself or your family down? ____
7. Trouble concentrating on things, such as reading the newspaper or watching television? ____
8. Moving or speaking so slowly that other people could have noticed. Or, the opposite – being so fidgety or restless that you have been moving around a lot more than usual. ____
9. Thoughts that you would be better off dead or of hurting yourself in some way? ____

Part 8 – Read:

1. **Now, thinking of the past month, have you had any thoughts that you would be better off dead or hurting yourself in some way? (If yes) How have you acted on these thoughts?** *Probes: Please describe to me what happened. Were you with others? [Probe to see if the accident was intentional].*

Section C: *For <u>all</u> Women*

Part 9 – Read:

1. **In general, would you say that your mental health is:** *(Read options)*
   ___ Excellent
   ___ Very Good
   ___ Good
   ___ Fair
   ___ Poor
   ___ *Unknown/Refused*

2. **In general, would you say that your physical health is:** *(Read options)*
   ___ Excellent
   ___ Very Good
   ___ Good
   ___ Fair
   ___ Poor
   ___ *Unknown/Refused*

Part 10 – Read:

1. **Have you applied for disability for a mental illness?**

   1a. If yes, which illness?______________________________

   1b. Did you receive disability for it?_________

Part 11 – Read:

1. **In my community, people take care of their emotional problems on their own; they do not seek professional mental health services**. *(Read options)*
   ___ 1=Strongly disagree
   ___ 2=Disagree
   ___ 3=Agree
   ___ 4=Strongly agree

2. **I feel confident that I could find a therapist that is understanding and respectful of my ethnicity/culture**. *(Read options)*
   ___ 1=Strongly disagree
   ___ 2=Disagree
   ___ 3=Agree
   ___ 4=Strongly agree

***Section C:** For <u>all</u> Women*

Part 12 – Read:

1. **What is the number of days that you experienced hot flashes in the past two weeks?**
   ___ 0 Days *(Skip to next section)*
   ___ 1-5 Days
   ___ 6-8 Days
   ___ 9-13 Days
   ___ Everyday
   ___ *Unknown/Refused*

2. **How many hot flashes do you have a day, on average?** ____

Part 13 – Read:

1. **In the past 3 months, did you have eating binges or times when you ate a large amount of food within a 2 hour period?**
   ___ Yes *(move to question 1a and 1b)*
   ___ No *(skip to next section)*

1a. Why do you think you did that? ___________________

1b. During these binges, do you feel that your eating was out of control?
___ Yes
___ No

Section C: *For <u>all</u> Women*

Part 14 – Read:

1. **Do you presently use recreational drugs other than what is prescribed to you?**
   ___ Yes *(move to question 1a-1h)*
   ___ No *(skip to question 2)*

   1a. At what age did you start using them? ____________________

   1b. What? *(current use)* ___________________

   1c. How often? *(current use)* _____________________

   1d. Have you ever felt that you ought to cut down on your drug use?
   ___ No ___ Yes

   1e. Can you get through the week without using?
   ___ No ___ Yes

   1f. Have people annoyed you by criticizing your drug use?
   ___ No ___ Yes

   1g. Have you felt bad or guilty about your drug use?
   ___ No ___ Yes

   1h. Have you ever used drugs first thing in the morning to steady your nerves?
   ___ No ___ Yes

2. **Do you presently drink any alcoholic beverage?**
   ___ Yes *(move to question 2a-2h)*
   ___ No *(skip to next section)*

   2a. At what age did you start drinking? ____________________

   2b. What? *(current use)* ___________________

   2c. How often? *(current use)* _____________________

   2d. Have you ever felt that you ought to cut down or your drinking?
   ___ No ___ Yes

   2e. Can you get through the week without drinking?
   ___ No ___ Yes

   2f. Have people annoyed you by criticizing your drinking?
   ___ No ___ Yes

   2g. Have you felt bad or guilty about your drinking?
   ___ No ___ Yes

   2h. Have you ever used alcohol first thing in the morning to get rid of a hangover?
   ___ No ___ Yes

Section C: *For* *all* *Women*

Part 15 – Only ask questions that were not answered in Section A:

1. **Before age 18, were you ever physically punished or beaten by a parent, caretaker, or teacher so that: you were very frightened; or thought you would be injured; or you received bruises, cuts, welts, lumps or injuries?**
___Yes *(move to question 1a and 1b)*
___No *(skip to question 2)*
1a. At what age(s)? ______ *(beginning age; if repetitive, indicate age range)*

1b. How many times? *(Circle number of times before age 18)*

1 / 2 – 4 / 5 - 10 / More than 10

(Probes: Tell me about that. Who did it? Was it recurrent? Did somebody else know this was happening?)

2. **Have you ever been in any situation in which you were seriously injured, or have you ever been in any other situation in which you feared you might be seriously injured or killed?**
(Other than what you described before age 18 years above)
___Yes *(move to question 2a and 2b)*
___No *(skip to question 3)*

2a. At what age(s)? ______ *(beginning age; if repetitive, indicate age range)*

2b. How many times? *(Circle number of times over lifetime)*

1 / 2 – 4 / 5 - 10 / More than 10

(Probes: Tell me about that. Were you alone? How long did it last? What was the scariest moment?)

3. **Has anyone, including family members or friends, ever attacked you with a gun, knife or some other weapon?**
___Yes *(move to question 3a and 3b)*
___No *(skip to question 4)*

3a. At what age(s)? ______ *(beginning age; if repetitive, indicate age range)*

3b. How many times? *(Circle number of times over lifetime)*

1 / 2 – 4 / 5 - 10 / More than 10

(Probes: Tell me about that. Were you alone? How long did it last? What was the scariest moment? Was it family or friends? Did other family/friends know? If other people knew, what was done to prevent it from happening again?)

Section C: For all Women

4. **Have you ever seen someone seriously injured or killed?**
   ___Yes *(move to question 4a and 4b)*
   ___No *(skip to question 5)*

   4a. At what age(s)? ______ *(beginning age; if repetitive, indicate age range)*

   4b. How many times? *(Over lifetime)*

   1 / 2 – 4 / 5 - 10 / More than 10

(Probes: Tell me about that. Were you alone? What was your relationship to them? Were you worried about yourself? What was the scariest moment?)

5. **Has anyone ever made you have intercourse or oral or anal sex against your will?**
   ___Yes *(move to question 5a and 5b)*
   ___No *(skip to question 6)*

   5a. At what age(s)? ______ *(beginning age; if repetitive, indicate age range)*

   5b. How many times? *(Over lifetime)*

   1 / 2 – 4 / 5 - 10 / More than 10

(Probes: Tell me about that. Who did it? Was it recurrent? Did somebody else know this was happening?)

6. **Has anyone ever touched private parts of your body, or made you touch theirs, under force or threat?**
   ___Yes *(move to question 6a and 6b)*
   ___No *(skip to question 7)*

   6a. At what age(s)? ______ *(beginning age; if repetitive, indicate age range)*

   6b. How many times? *(Circle number of times over lifetime)*

   1 / 2 – 4 / 5 - 10 / More than 10

(Probes: Tell me about that. Who did it? Was it recurrent? Did somebody else know this was happening?)

Section C: *For <u>all</u> Women*

7. **Have you ever been emotionally abused or neglected (for example, being frequently shamed, embarrassed, ignored, or repeatedly told that you were "no good")?**
   ___Yes *(move to question 7a and 7b)*
   ___No *(skip to question 8)*

   7a. At what age(s)? ______ *(beginning age; if repetitive, indicate age range)*

   7b. How many times? *(Circle number of times over lifetime)*

   1 / 2 – 4 / 5 - 10 / More than 10

Probes: Ask if it was emotional abuse (verbal insults) or physical neglect (physical needs ignored) - Tell me about that. Who did it? Was it recurrent? Did somebody else know this was happening?

8. **Up to the age of 18, did you ever live without at least one of your biological parents?**
   ___Yes *(move to question 8a)*
   ___No

   8a. At what age(s)? ______

   8b. For how long?

(Probes: Tell me about that. What led to this? Who did you live with? Was this a good experience for you?

After this question, remember to:

- **Thank the participant for sharing (noting how difficult it may have been to open up)**
- **Ask whether the participant has any questions or final thoughts to add**
- **Mention focus group; Process gift card/gift log; provide MH resources**
